# Point-of-care test for rapid assessment of blood lithium levels in women with bipolar disorder during perinatal period

**DOI:** 10.1192/j.eurpsy.2022.420

**Published:** 2022-09-01

**Authors:** M.L. Imaz, M. Torra, M. Martin, I. Aliart, R. Martin-Santos, E. Vieta, L. Garcia-Esteve

**Affiliations:** 1 Hospital Clinic, Unit Of Perinatal Mental Health Clínic-bcn. Department Of Psychiatry And Psychology, Barcelona, Spain; 2 Hospital Clinic, Pharmacology And Toxicology Laboratory, Biochemistry And Molecular Genetics Service, Biomedical Diagnostic Center (cbd), Barcelona, Spain; 3 Hospital Clinic, Psychiatry And Psychology, Barcelona, Spain

**Keywords:** Perinatal period, Lithium, Point of care test, bipolar disorder

## Abstract

**Introduction:**

Determination of lithium levels in serum has become a standard of care due to its narrow therapeutic rang, thus an immediate test for determination of blood lithium may contribute to minimize toxicit, to avoid relapse and to ensure treatment adherence. This is particularly relevant during pregnancy and early postpartum because pharmaokinetic changes in renal physiology.

**Objectives:**

The aim of this study is verify Medimate point-of-care method performance and systematically compare it with the routine laboratory measurement of lithium.

**Methods:**

This cross-sectional method comparison study was conductec in the Unit of Perinatal Mental Health in the Hospital Clinic of Barcelona. Pearson and Bland-Altman analyses were performed to assess the accuracy, precision and correlation between the capillary electrophoresis technology (Medimate MiniLab) and the ion selective electrode (ISE) potentiometry method (AVL 9180).

**Results:**

Twenty-five women with bipolar disorder in treatment with lithium during perinatal period were enrolled, corresponding to 75 blood specimens for analyses. Correlation (r), mean difference (bias), and 95% limit of agreement (LOA) of the point-of-care method [r=0.917; bias 0.0021 (95% LOA; 0.440, 0.619) mEq/L], showed that difference between ISE method and capillary electrophoresis technology was not statistically significant.

**Conclusions:**

Considering the practicality, the microchip capillary electrophoresis technology provides a simple and highly affordable way of measuring lithium levels in a single drop of blood outside the clinical laboratory. The Medimate point-of-care system (POC) appears well adapted for the rapid and specific detection of lithium as an alternative to the current ISE procedure.

**Disclosure:**

No significant relationships.

